# Effectiveness of Psychoeducational Interventions for Improving Symptoms, Health-Related Quality of Life, and Psychological well Being in Patients with Stable Angina

**DOI:** 10.2174/157340308783565393

**Published:** 2008-02

**Authors:** M McGillion, H Arthur, J.C Victor, J Watt-Watson, T Cosman

**Affiliations:** 1 McMaster University, Faculty of Health Sciences, 1200 Main St. W. (HSc 2J20A), Hamilton, ON, Canada, L8N 3Z5; 2 University of Toronto, 155 College Street, Toronto, ON, Canada, M5T 1P8; 3 Hamilton Health Sciences, 237 Barton Street East, 3rd Floor- Lower North Room L 303, Hamilton, ON, Canada, N3L 2Y6

**Keywords:** Stable angina, psychoeducation, angina symptoms, health-related quality of life, psychological well-being, meta-analysis.

## Abstract

Several primary trials report the adjunctive value of psychoeducational interventions for improving stable angina symptoms, health-related quality of life (HRQL) and psychological well-being; however, few high-quality meta-analyses have examined the overall effectiveness of these interventions. We used meta-analysis in order to determine the effectiveness of psychoeducational interventions for improving symptoms, HRQL and psychological well-being in stable angina patients. Seven trials, involving 949 participants total were included. Those who received psychoeducation experienced nearly 3 less angina episodes per week, delta (Δ)= -2.85, 95% CI, -4.04 to -1.66, and used sublingual (SL) nitrates approximately 4 times less per week, Δ= -3.69, 95% CI -5.50 to -1.89, post-intervention (3-6 months). Significant HRQL improvements (Seattle Angina Questionnaire) were also found for physical limitation, Δ= 8.00, 95% CI 4.23 to 11.77, and disease perception, Δ= 4.46, 95% CI 0.15 to 8.77, but CIs were broad. A pooled estimate of effect on psychological well-being was not possible due to heterogeneity of measures. Psychoeducational interventions may significantly reduce angina frequency and decrease SL nitrate use in the short-term. These encouraging results must be interpreted with caution due to heterogeneity in methods and small samples. Larger, robust trials are needed to further determine the effectiveness of psychoeducation for stable angina management.

## INTRODUCTION

Chronic stable angina (CSA) pectoris is a ubiquitous and cardinal symptom of ischemic heart disease (IHD) characterized by pain or discomfort in the chest, upper abdomen, back, arm(s), shoulders, neck, jaw and/or teeth [1]. Angina is considered stable if symptoms are experienced over several weeks in the absence of major deterioration [2]. Stable angina symptomatology can vary depending on factors such as demand for increased myocardial blood flow, stress, emotions, diet and weather, and may range from Canadian Cardiovascular Society (CCS) Class I to Class III angina [3].

Evidence suggests that greater than 64% of patients with CSA are taking more than 1 cardiovascular drug to treat their symptoms [4]. Despite this, angina persists in more than 90% of patients [5]. CSA is distressing, with a well-documented, major negative impact on health-related quality of life (HRQL). Individuals with CSA often live with recurrent pain episodes, poor general health, impaired role functioning, activity restriction, and reduced capacity for self-care [6-18]. These patients are also at risk for acute myocardial infarction, congestive heart failure, atrial fibrillation, and stroke [19], as well as increased risk of cardiovascular-related mortality or hospitalization (men: RR 1.62, women: RR 1.48) [20].

Prevalence data from 1999-2002 suggest that more than 6,500,000 Americans may be living with CSA [21]. In Scotland, CSA prevalence (April 2001-March 2002) has been estimated at 28/1000 men and 25/1000 women [22]. While the prevalence of CSA in Canada has not been studied directly, 36% of Canadians aged 35 – 64 years who reported having a diagnosis of heart disease in a National Population Health Survey (n = 345,000) reported angina symptoms and related daily activity restriction^a^. The Canadian Laboratory Centre for Disease Control also found that in 1995, 16% of all physician visits related to heart disease in Canada (29.6 million visits) involved a complaint of angina^b^.

CSA also imposes numerous direct and indirect societal costs. A recent economic pilot study by McGillion *et al*. (2006, [published abstract]) found that the total median annualized societal cost of CSA was $12,615 (Canadian) per person (2003 – 2005); indirect costs accounted for 2/3 of the total CSA-related cost of illness [23] In the UK, the direct cost of CSA in 2000, including prescriptions, admissions, outpatient referrals, and procedures, was estimated at ₤669, 000, 000, accounting for 1.3% of the total National Health Service expenditure [24]. 

Considering the available data on prevalence, impact, health service utilization and costs, CSA is a major and debilitating health problem. National guidelines are readily available that summarize the best evidence about pharmacological and percutaneous coronary interventions for improvement of angina symptoms and related HRQL [25, 26]. While several primary studies report the value of psychoeducational interventions as an adjunctive means of improving CSA symptoms, HRQL and psychological well-being [27-33], no high-quality syntheses, using meta-analytic techniques, have been conducted to examine the effectiveness of these particular interventions. Psychoeducational interventions are multi-modal treatment packages that employ learning materials and cognitive-behavioural strategies to achieve changes in knowledge and behaviour for effective disease self-management [34]. They target day-to-day problems that patients encounter such as pain, fatigue, decreased mobility and endurance, anxiety and stress [34]. Over a course of several days or weeks, an array of self-management techniques are taught that patients can rehearse and incorporate into their daily routines, such as a) safe exercise habits, b) energy conservation, pacing, and sleep quality enhancement, and c) communication and decision making skills. Psychoeducation programs can vary, ranging from individual to group-based formats, with either prescriptive or flexible curricula. More flexible programs allow for a choice of self-management technique(s) that best suit one’s personality, lifestyle, ability, and confidence level [35]. Irrespective of format, a firm grounding in social, cognitive and/or behavioural theories is critical to the success of most psychoeducation programs [33-40]. These theories, such as Bandura’s Self-Efficacy Theory, target patients’, a) confidence to achieve optimal functioning, b) acceptance of their chronic illness-induced limitations, and c) more adaptive ways of thinking, feeling and behaving [33-40]. A recent review of psychoeducation trials across divergent chronic illness populations found that adherence to self-efficacy enhancing principles consistently resulted in improved knowledge, performance of self-management behaviours, and various aspects of physical and emotional functioning such as exercise capacity and mood status [35].

To date, most psychoeducation programs designed specifically for patients with CSA have targeted confidence in angina self-management, reduction of angina symptoms and related sublingual (SL) nitrate use, as well as improvements in HRQL. Yet, existing reviews of these programs are either narrative-based [41], or have examined the effectiveness of a broad array of psychological interventions, with or without conventional cardiac rehabilitation, for general coronary artery disease populations [42].

## OBJECTIVES

To determine the effectiveness of psychoeducational interventions for improving angina symptoms, HRQL, and psychological well-being for patients with CSA.

## CRITERIA FOR SELECTION OF STUDIES INCLUDED IN THIS REVIEW

### Study Designs

All published and unpublished randomized controlled trials of psychoeducational interventions delivered by a trained professional in individual or group formats, with parallel designs; follow up period varied. Non-randomized studies and single-group design studies were excluded.

### Participants

Adult outpatients of all ages with CAD and Canadian Cardiovascular Society Class I – III angina, experiencing stable symptoms for at least 6 months.

### Types of Interventions and Controls

Psychoeducational interventions employing a combination of cognitive and behavioural angina self-management techniques such as energy conservation, pacing, anxiety and stress management or counselling, exercise, dietary planning, safe SL nitrate use, and relaxation training. Controls received routine or usual care and were not exposed to the intervention during the study period.

### Outcomes Measures

1. Angina symptom profile including angina frequency and duration, and related SL nitrate use
2. Self-reported HRQL
3. Psychological well-being, reflected by anxiety, stress and/or depression

## SEARCH METHODS FOR IDENTIFICATION OF STUDIES 

We searched the Cochrane Central Register of Controlled Trials, MEDLINE, PubMed, CINHAL, EMBASE, Proquest Dissertation Abstracts, Psychinfo and HealthStar, Jan 1990 – Oct 2006, using combinations of key medical subject heading (MeSH) terms including chronic stable angina, angina pectoris, psychoeducation, psychosocial factors, stress/prevention and control, patient education, education randomized controlled trials, and clinical trials. We also conducted hand searches of relevant journals, proceedings of major conferences, and secondary references; experts in the field were consulted for additional sources. We planned to contact authors where possible to obtain missing information. Our search strategy was critiqued and replicated by an external information specialist to ensure comprehensiveness.

## METHODS

### Final Selection of Trials

Three reviewers reached consensus on all trials to be included in this analysis by reviewing the titles, abstracts and reports of all trials according to the inclusion criteria specified *a priori*; individual trial results were not considered during this process.

### Data Extraction and Appraisal of Methodological Quality

Two reviewers participated in independent extraction of process and outcome data from each trial according to a standardized format used in a prior review [41]. The quality of included trials was assessed by three reviewers with respect to sample size, generation of randomization sequence, allocation concealment, standardized intervention delivery, reliability and validity of measurement instruments and response rate (RR), blinding of outcome assessment, and examination of group differences.

### Data Synthesis and Analysis

All outcomes examined in this study were continuous in nature. For all relevant outcome data, weighted mean differences (WMD) and associated 95% confidence intervals were calculated [43] using *Comprehensive Meta-Analysis Version 2, © Biostat. Inc*. and verified using SAS/STAT^®^ SOFTWARE. WMDs were calculated *via* the inverse variance method wherein the weight for each primary trial was determined by the inverse of the variance of its respective effect estimates [43]. Mean change scores were taken from the results of each study except one (Payne *et al*. 1994), in which mean change in angina frequency had to be estimated from graphical output. Standard deviations for mean change scores were taken directly from the results where available, or calculated using the mean change score, the test-statistic (e.g. F value) and the sample size. The follow-up period for relevant outcomes was short-term (up to 12 weeks) for most trials (Bundy *et al*., 1994; Gallacher *et al*., 1997; Lewin *et al*., 1995; Payne *et al*., 1994; McGillion *et al*., 2006), and extended to 24 weeks in one trial (Lewin *et al*., 2002). Therefore, sensitivity analyses for all outcomes were conducted including and excluding the maximum follow-up period; length of follow-up period did not significantly affect the direction of pooled estimates of effects or statistical significance.

With respect to angina symptom profile, one trial (McGillion *et al*. 2006 [published abstract]) did not use a symptom diary. Instead, the authors employed the Seattle Angina Questionnare (SAQ) [44] to collect 4 week angina frequency and nitrate use on 6 point scales where the response categories were: 1 = 4 or more times per day; 2 = 1 to 3 times per day; 3 = 3 or more times per week, but not every day; 4 = 1 to 2 times per week; 5 = less than once a week; 6 = none over the past 4 weeks; we had access to these original raw data. To enable the calculation of mean differences in angina frequency and nitrate use for meta-analysis, participants’ responses were recoded as weekly estimates where: 1 = 28 times per week; 2 = 14 times per week; 3 = 4 times per week; 4 = 2 times per week; 5 = 0.5 times per week; and 6 = 0 times per week. The original data and the recoded data were analyzed for congruency in testing the association between the intervention group and the placebo group. Weighted mean differences were then calculated both including and excluding this trial (McGillion *et al*. 2006) to examine if these approximations changed the overall conclusion; no impact on the significance of the results was found. 

## DESCRIPTION OF STUDIES

Eight trials, conducted in 7 countries, between 1994 and 2007, and involving 1,009 patients were identified for possible inclusion. One study was excluded that examined the impact of group psychological treatment on patients with non-ischemic chest pain [45]. Six of the included trials reported use of an isolated psychoeducational intervention with components designed to enhance patients’ perceived confidence and skills to manage angina symptoms (Bundy *et al*., 1994; Gallacher *et al*., 1997; Lewin *et al*., 1995; 2002; Payne *et al*., 1994; McGillion *et al*., 2006). Control groups received usual medical and/or nursing care as described; no controls were exposed to the intervention during the study period. One trial included a standardized medication regimen for both treatment and control groups (Ma and Teng, 2005).

Details of each included trial are presented in Table **1**. Five trials (Bundy *et al*., 1994; Gallacher *et al*., 1997; Lewin *et al*., 1995; Payne *et al*., 1994; McGillion *et al*., 2006) tested small-group interventions (6-15 patients) employing varying combinations of educational materials, planned exercise and cognitive-behavioural techniques targeted at lifestyle and symptom self-management, relaxation training or stress reduction, or enhancement of physical activity. Intervention duration, format, and process varied. Two trials tested interventions with content similar to the group-based interventions but on an individual basis (Lewin 2002; Ma and Teng 2005). All trials included baseline assessment of participant characteristics and outcomes prior to randomization. Most trials used symptom diaries to measure angina symptom profile and related SL nitrate use; objective measures of ischemia were less often used. Subjective measures were also most often used to examine HRQL and psychological well-being. Data pertinent to this review were collected up to 24 weeks following baseline (Lewin *et al*., 2002).

## METHODOLOGICAL QUALITY

Sample sizes ranged from 29 to 452; two trials included power analyses to support sample size (Lewin *et al*., 2002; McGillion *et al*., 2006). The method of randomization was unclear in three trials (Bundy *et al*., 1994; Lewin *et al*., 1995; Ma and Teng, 2005); stated randomization methods included sequential envelopes (Gallacher *et al*., 1997), alternating weekly cohorts (Payne *et al*., 1994), external randomization list (Lewin *et al*., 2002) and centrally-controlled computer-generated randomization (McGillion *et al*., 2006). Strategies used to preserve concealment of allocation sequence and blinding of outcome assessors were addressed in two trials (Lewin *et al*., 2002; McGillion *et al*., 2006). To varying degrees, all trials examined group differences in key baseline characteristics and pretest scores, and response rates across measurement occasions ranged from acceptable to excellent (67% - 100%). Most trials used a patient diary to record angina frequency, duration, intensity and SL nitrate use; the reliability and validity of these tools was not addressed. When measured, HRQL and aspects of psychological well-being were captured using well-established, reliable and valid measures. Only one trial addressed methods used to ensure standardized intervention delivery and adherence to intervention protocol (McGillion *et al*., 2006). Across trials, intervention delivery was limited to a single site; intervention formats, duration, and processes were variable. The results of two trials were applicable to men only (Gallacher *et al*., 1997; Payne *et al*., 1994). Based on our review of methodological quality, the majority of available trials are likely susceptible to a number of biases due the following potential threats to validity: inadequate power (sample size bias), unclear allocation concealment (selection bias) and blinding of outcome assessment (ascertainment bias), inadequate experimental controls (co-intervention), and unknown reliability and validity of measures (insensitive measure bias) [46-51].

## RESULTS

**Fig. (1):**
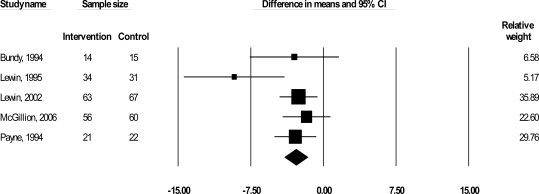
WMD in frequency of angina episodes per week.

**Fig. (2):**
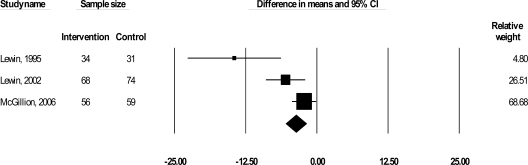
WMD in sublingual nitrate usages per week.

**Fig. (3):**
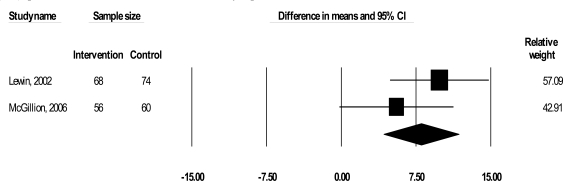
WMD in angina-induced physical limitation (SAQ-PL) scores (disease-specific health-related quality of life).

**Fig. (4):**
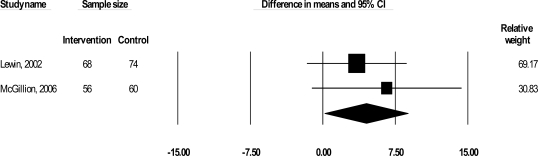
WMD in disease perception (SAQ-DP) scores (disease-specific health-related quality of life).

Seven trials, involving 949 CSA patients, were included in this review. It was not possible to include results from two trials (Gallacher *et al*., 1997, n = 452; Ma and Teng 2005; n = 100) in any pooled estimates of effect due to the heterogeneity of their measures and analyses. A summary of the results is presented in Table **2** by outcome, and in sequential order, starting with symptom profiles, followed by HRQL and psychological well-being. Figs. **1-4** present the meta-analysis graphs for significant outcomes respectively. All results pertain to pooled short-term effects, given the maximum length of follow-up of 24 weeks.

### Angina Symptom Profile

Five trials (Bundy *et al*., 1994 ; Lewin *et al*., 1995; 2002; Payne *et al*., 1994; McGillion *et al*., 2006) reported on angina frequency (383 patients), which we examined according to mean change in number of angina episodes per week. Results suggested a significant short-term reduction in angina frequency by 2.85 episodes per week, *delta* (Δ) = -2.85, 95% confidence interval (CI), -4.04 to -1.66, p < 0.001 (Fig. **1**). Angina duration was reported in three trials (224 patients) (Bundy *et al*., 1994; Lewin *et al*., 1995; 2002) and was examined according to mean change in number of minutes per angina episode. Although results suggested a significant short-term 1/2 minute reduction in angina per episode, Δ = -.51, 95% CI, -.81 to -0.21, p= 0.001, Bundy *et al*.’s study with a small sample (n = 29) carried 99% of the weight in this analysis due to its small standard deviation. We removed this study from the duration analysis given that a) it was influencing this meta-analytic result so heavily and, b) low variance in scores is unusual for small samples; once removed, the direction of the observed effect remained unchanged yet the summary difference in means was no longer significant: Δ = -5.86, 95% CI, -13.97 to 2.25, p= 0.001.

SL nitrate use was also reported in three trials (310 patients) (Lewin *et al*., 1995; 2002; McGillion *et al*. 2006) and was examined according to mean change in number of nitroglyercine uses (one use included up to 3 sprays) per week. Psychoeducational intervention resulted in a significant reduction in nitrate use by 3.69 times per week: Δ = -.3.69, 95% CI, -.5.50 to -1.89, p< 0.001 (Fig.**2**). 

We could not find any clear *a priori* evidence of what constitutes clinically significant reductions in angina symptoms and/or sublingual nitrate use. We therefore used Cohen’s *d* formula for standardized mean differences between groups (*d* = M_1_ - M_2_ / σ) to calculate effect size (ES) as an initial indicator of clinical significance [52]. Effect size estimates for angina frequency and SL nitrate use were 0.49 and 0.53 respectively. 

### Health-Related Quality of Life

Two trials examined the impact of their interventions on self-reported HRQL (Lewin *et al*., 2002; McGillion *et al*., 2006) with the disease-specific SAQ (267 patients) [43]. The SAQ quantifies five clinically relevant domains of disease-specific HRQL including physical limitation (PL), anginal stability (AS), anginal frequency (AF), treatment satisfaction (TS), and disease perception (DP); no overall HRQL summary score is derived [43]. We excluded AF and AS from this analysis as raw SAQ AF and AS-related data (from McGillion *et al*.’s trial) were factored into our symptom profile analyses. As shown in Figs. **3** and **4**, results suggested significant improvements in PL [Δ = 8.00, 95% CI, 4.23 to 11.77, p< 0.001] and DP [Δ = 4.46, 95% CI, 0.15 to 8.77, p= 0.042], but CIs were wide; effect sizes were 0.51 and 0.26 respectively. No significant improvement in TS was found.

### Psychological Well-Being

Three trials reported on psychological well-being using the Centre for Epidemiologic Studies Depression Scale and the Spielbeger State-Trait Personality Inventory (Payne *et al*. 1994), the Derogatis Stress Profile (Gallacher *et al*. 1997) and the Hospital Anxiety and Depression Scale (Lewin *et al*. 2002) respectively; a pooled estimate of effect on psychological well being was not possible due to the heterogeneity of these measures.

## DISCUSSION

In this review we have appraised and summarized the results of 7 trials of psychoeducational interventions for CSA management, conducted in 6 different countries, in a variety of outpatient, hospital and community-based settings. The search strategy used to identify these trials was as comprehensive as possible for CSA-specific psychoeducational interventions, without language restrictions. 

The first outcome examined was angina symptom profile. Pooling the results of 5 of the included trials suggested that psychoeducational interventions significantly reduce angina frequency and nitrate use, and enhance aspects of disease-specific HRQL in the short term. Angina frequency and nitrate use were decreased by approximately 3 and 4 times per week respectively. These results are encouraging given the high levels of perceived psychological burden and reports of poor quality of life associated with continued angina symptoms [6-18, 53, 54]. Effect size estimates for angina frequency and nitrate use were 0.49 and 0.53 respectively, suggesting that psychoeducation offers moderate short-term positive effects on these outcomes (Table **2**). Although our pooled estimate of effect on SL nitrate use may be clinically significant in the context of the primary trials reviewed, overall reductions in nitrate use are not always desirable. Many patients benefit from chronic therapy *via* oral and/or transcutaneous long-acting nitrate preparations (with nitrate-free intervals to minimize tolerance), in addition to the routine use of sublingual nitrates for acute angina episodes [55]. Like short-acting preparations, long-acting nitrates decrease cardiac workload and oxygen demand by reducing left ventricular (LV) preload and afterload. In addition, they optimize redistribution of blood flow to ischemic subendocardium by decreasing LV end diastolic pressure and dilating epicardial vessels [55]. 

Education about nitrates in the included trials was limited to the proper administration of SL nitrates in order to manage acute angina. This limitation points to the need to incorporate and examine the effectiveness of more comprehensive approaches to nitrate-based angina management in future psychoeducation trials; recent evidence suggests that a) patients are frequently confused and/or uninformed about the principles of short and long-acting nitrate self-administration [38]. 

The second outcome was self-reported HRQL, which was measured in 2 trials (Lewin *et al*., 2002; McGillion *et al*., 2006) using the disease-specific SAQ. Combined results of these trials suggested that psychoeducational interventions yield significant, small (ES = 0.26) to moderate (ES = 0.51) short-term improvements in the perception of the overall impact of heart disease on HRQL (SAQ-DP), and angina-induced physical limitations (SAQ-PL) respectively (Table **2**). While these results were statistically significant, CIs were wide. In addition, the WMDs in SAQ-DP and SAQ-PL scores were 4.46 and 8.00 respectively; prior work has established that minimum 5-8 point improvements across SAQ subscales (except the SAQ-AS scale, for which larger changes are clinically meaningful) are required to reflect clinically meaningful change in disease-specific HRQL for angina patients [15,44,56,57]. Therefore, only the pooled estimate of effect on angina-induced physical limitations may be of clinical significance. 

The similar samples of the 2 trials, as well as their relatively equal weighting (49.08%; 50.92%) and variance in SAQ-PL scores (Fig. **3**), suggests that differences in the strength of their respective interventions may have been a key factor in their clinically sub-optimal, combined impact on physical functioning. One trial tested the effects of community group-based psychoeducation (McGillion *et al*. 2006), and observed greater positive short-term impact on angina-induced physical limitations than the other trial, which examined individual counseling in a clinic setting, with                 supportive telephone follow-up (Lewin 2002). However, discrepancy in the end-points of these studies signals caution in making such inferences about the relative strengths of their interventions. In addition, although there was clear overlap in content, the interventions in these studies were somewhat heterogeneous with respect to duration, format and process; the degree to which this heterogeneity negatively affected statistical and/or clinical significance is unclear. 

With respect to disease perception, it appeared that the individual-based clinic intervention had a more uniform impact on disease perception scores (SAQ-DP) than the group-based intervention, but to a less-positive degree on average (Fig. **4**). High variance in scores was therefore likely a main contributor to the observed pooled effect on disease perception, in addition to any diluting impact that intervention heterogeneity may have exerted.

Neither trial included in HRQL analyses reported significant positive effects on treatment satisfaction; not surprisingly, the pooled effect on treatment satisfaction scores (SAQ-TS) was also insignificant. Beyond variance in scores and intervention differences, statistically insignificant results for treatment satisfaction (TS) at the individual trial level were likely primarily driven by the psychometric properties of the SAQ-TS scale. This scale is comprised of 3 items oriented toward patient satisfaction with physician care [44]. Despite their differences, the interventions in both trials were delivered by nurses, not physicians, and the respective short-term end points of these trials likely did not allow sufficient time for potential improvements in patient-physician rapport.

The third outcome of this review was psychological well-being. While a number of reviews have suggested that broader psychological interventions are of clinical value for patients with heart disease, there is insufficient evidence to make recommendations since pooled estimates of the effect of psychoeducation on psychological well-being were not possible.

Overall, though the meta-analytic results of this review appear somewhat promising for the outcomes that were amenable to pooled estimates of effect, they must be interpreted with a high level of caution. Confidence intervals, for HRQL in particular, were wide. The methodological quality of the included trials ranged from good to poor. Comprehensiveness in examining baseline group differences varied, sample sizes were generally small, and trial reports often lacked detail with respect to allocation concealment, outcome assessment, reliability and validity of symptom-related measures, standardized intervention delivery, and experimental controls. Moreover, only data from selected trials could be pooled to estimate combined effects on angina symptom profile and HRQL due to the heterogeneity of a number of measures used. Finally, interventions across trials were heterogeneous with respect to duration, format (e.g. individual versus group-based) and process; intervener credentials and substantive content also varied.

### Implications

Key questions about the effectiveness of psychoeducational interventions for patients with CSA remain unanswered. A common and seemingly critical element among these interventions was the provision of a variety of                 supports to enhance patients’ confidence and skills for angina-self-management. Yet, the ideal intervention design that would yield maximal and replicable benefit for these patients is not known. This next step is critical to developing psychoeducation as a potential mainstay of adjunctive treatment of stable angina. Major and reliable improvements in HRQOL, self-efficacy, and illness-related costs have consistently been reported in other complex chronic populations (e.g. arthritis, inflammatory bowel disease, chronic non-cancer pain), once ideal intervention designs have been validated [40,58,59].

The sustainability of observed improvements in angina symptom profile and aspects of self-reported HRQL has also not been examined. A more comprehensive approach to educating participants about the principles of safe nitrate use, beyond the management of acute angina episodes, is also required. Each trial also used a single site to test their respective interventions. Therefore, individual trial results may                 be context-dependent and have limited generalizability. Robust multi-site trials with adequate power, standardized and replicable interventions, consistency in measurement, and longer-term follow-up are needed to avoid potential sources of bias and determine, more definitively, the effectiveness of psychoeducational interventions as an adjunctive means for improving CSA symptoms, HRQL and psychological well-being.

## SUMMARY

CSA is a major clinical problem that poses considerable societal burden and deleterious impact on HRQL. While pooled trial results suggest that psychoeducational interventions may have a positive, short term impact on angina symptom frequency, SL nitrate use, and aspects of self-reported HRQL, additional well-designed trials are required to determine both the magnitude and sustainability of the effect of these interventions for CSA patients.

## Figures and Tables

**Table 1.** Characteristics of Included Studies

Study	Bundy *et al*. 1994
Design	RCT
Relevant outcomes/definitions	Angina symptoms/severity, frequency, duration, intensity; exercise capacity/bicycle ergometer; anxiety and depression/HADS
Sample size (after LTF); participant characteristics; setting; country	N= 29; male and female CSA patients aged 46-63; unclear; Clwyd, UK
Measurement occasions; outcome measures: reliability and validity; RR	8 and 16 weeks post intervention; daily angina diary: not addressed, ergometer: not addressed, HADS: reliable and valid; 100%
Method of randomization	Unclear
Allocation concealment	Unclear
Intervention	Small group 7-week (1.5 hours weekly) program on stress, anger and lifestyle management, problem solving, cognitive control, relaxation training; clinical psychologist intervener
Blinding of outcome assessment	Unclear
Examination of group differences	Done
Notes	Inadequate discussion of intervention controls and description of sample
Study	Gallacher *et al*. 1997
Design	RCT
Relevant outcomes/definitions	Chest pain and discomfort/severity, frequency, duration, intensity; stress/DSP
Sample size (after LTF); participant characteristics; setting; country	N= 452; male CSA patients, <70 years of age, 87%; unclear; South Glamorgan, Wales
Measurement occasions; outcome measures: reliability and validity; RR	Baseline and 6 months post-intervention; 14-day diary of chest pain and discomfort: not addressed, DSP: reliable and valid; 87%
Method of randomization	Sequential groups of 8 envelopes (4 in each included in the intervention)
Allocation concealment	Unclear
Intervention	3 1-hour biweekly small group sessions on stress management and relaxation techniques; intervener not identified
Blinding of outcome assessment	Unclear
Examination of group differences	Done
Notes	Inadequate discussion of intervention controls; results generalizable to men only
Study	Lewin *et al*. 1995
Design	RCT
Relevant outcomes/definitions	Angina/severity, frequency, duration, intensity, nitrate use; disability/SIP; exercise tolerance/ECG treadmill test
Sample size (after LTF); participant characteristics; setting; country	N= 65; male and female CSA patients; hospital clinic setting; England
Measurement occasions; outcome measures: reliability and validity; RR	Baseline and immediate, 4 months, and 1 year post intervention; angina diary: not addressed, SIP: reliable and valid, ECG: reliable and valid; 87%
Method of randomization	Unclear
Allocation concealment	Unclear
Intervention	8-week combined small group/individual session rehabilitation program (2 mornings a week) featuring relaxation training, identification of mal-adaptive behaviours, coping strategies, goal setting; clinical psychologist and physiotherapist interveners
Blinding of outcome assessment	Unclear
Examination of group differences	Done- extensive
Notes	Inadequate discussion of intervention controls
Study	Payne *et al*. 1994
Design/Country	RCT
Relevant outcomes/definitions	Chest pain/frequency and intensity likert scales; Prevailing mood and psychological stress/ CES-D, STPI
Sample size (after LTF); participant characteristics; clinical setting; country	N= 43; male veteran CSA patients < 65 years of age; unclear; USA
Measurement occasions; outcome measures: reliability and validity; RR	Sessions 1 and 3, 1 and 6 months follow-up; chest pain likert scales; not addressed, CES-D and STPI: reliable and valid; 83% at 1 month, 67% at 6 months
Method of randomization	Based on alternating weekly cohorts
Allocation concealment	Unclear
Intervention	3-week small group angina management program, featuring cognitive stress management and relaxation techniques; intervener not identified
Blinding of outcome assessment	Unclear
Examination of group differences	Done- extensive
Notes	Whether allocation was random is unclear; results generalizable to men only
Study	Lewin *et al*. 2002
Design	RCT
Relevant outcomes/definitions	Anxiety and depression/HADS; Angina/angina frequency and nitroglycerine use; HRQL/SAQ
Sample size (after LTF); participant characteristics; setting; country	N= 130; patients with diagnosis of angina within 1 year, average age: treatment 66.74 (SD 9.37), controls 67.64 (9.01); outpatient clinic; England
Measurement occasions; outcome measures: reliability and validity; RR	Baseline and 6 months; angina diary: not addressed, HADS and SAQ: reliable and valid; 91% at 6 months
Method of randomization	Randomization by list held at a remote site
Allocation concealment	Person responsible for randomization list blinded to patients
Intervention	Individualized cognitive-behavioural disease management program featuring structured interview, patient-held workbook, audio-taped relaxation program, angina misconceptions, risk-factor assessment, lifestyle change, telephone support, education sessions; nurse intervener
Blinding of outcome assessment	Done
Examination of group differences	Done- extensive
Notes	
Study	McGillion *et al*. 2006
Design	RCT
Relevant outcomes/definitions	HRQL/Medical Outcome Study SF-36, SAQ
Sample size (after LTF); participant characteristics; setting; country	N=130; male and female CSA patients, average age 68 (SD 10.6); unclear; Canada
Measurement occasions; outcome measures: reliability and validity; RR	Baseline and 3 months; SF-36 and SAQ: reliable and valid; 87%
Method of randomization	Centrally-controlled computerized randomization
Allocation concealment	Centrally protected by computer
Intervention	Small group 6-week self-management program featuring self-efficacy, weekly goal setting, energy conservation, decision making, emotional responses, symptom management techniques, patient workbook, meaning of angina, safe exercise, emergency management, protocol and external auditor used to ensure standardized delivery; nurse intervener
Blinding of outcome assessment	Done
Examination of group differences	Done- extensive
Notes	Contact information: Dr. Michael McGillion, University of Toronto Email: michael.mcgillion@utoronto.ca
Study	Ma and Teng 2005
Design	RCT
Relevant outcomes/definitions	Anxiety and depression/anxiety and depression charts; angina/24 hour ambulatory ECG
Sample size (after LTF); participant characteristics; setting; setting	N= 100; male and female CSA patients, age range/average not given; recruited in cardiology inpatient setting during medical management of angina; China
Measurement occasions; outcome measures: reliability and validity; RR	Unclear; anxiety and depression charts: not addressed, 24 hour ambulatory ECG: reliable and valid; 100%
Method of randomization	Unclear
Allocation concealment	Unclear
Intervention	Standardized drug treatment applied to both groups: nitrates, anti-coagulants; Individualized 8- week Beck’s cognitive intervention for treatment group featuring angina review; negative cognitions identification, angina misbeliefs and related home-based work; physician intervener
Blinding of outcome assessment	Done
Examination of group differences	Done
Notes	Translated by Fang Zhang-Helwig, University of Toronto; measurement occasions unclear

*NB*: CES-D: Centre for Epidemiological Studies Depression Scale; DSP= Derogatis Stress Profile; ECG= electrocardiogram; HADS= Hospital Anxiety and Depression Scale; HRQL= health-related quality of life; RCT= randomized controlled trial; RR= response rate; SAQ= Seattle Angina Questionnaire; SD= standard deviation; SIP= Sickness Impact Profile; STPI= Spielberger State-Trait Personality Inventory.

**Table 2.** Results

Variable	Difference in Change	95% CI for Difference	p-value	Effect Size *(Cohen’s d)*
***Angina Symptom Profile***				
*Angina Frequency*	-2.85	[-4.04, -1.66]	<0.001	0.49
*Angina Duration* *Adjusted*	-0.51 -5.86	[-0.81, -0.21] [-13.97, 2.25]	0.001 0.157	0.31 0.20
*Nitroglycerine Use*	-3.69	[-5.50, -1.89]	<0.001	0.53
***Self-Reported HRQL***				
*SAQ – DP*	4.46	[0.15, 8.78]	0.042	0.26
*SAQ – PL*	8.00	[4.23, 11.77]	<0.001	0.51
*SAQ - TS*	2.76	[-1.47, 6.99]	0.201	0.17
*Psychological Well-Being* Pooled estimates of effect not possible				

*NB*: DP= disease perception; HRQL= health-related quality of life; PL= physical limitation; SAQ= Seattle Angina Questionnaire; TS= treatment satisfaction.

## Footnotes

a Statistics Canada, Health Services Division. Unpublished vital statistics standard tables for 1996, Special tabulations. In: Health Canada: Statistical report on the health of Canadians.

b Intercontinental Medical Statistics Canada. Heart Disease and stroke in Canada. In: Unpublished Data. 1995, Health Canada, Health Promotion Branch- LCDC.
